# Stability of Hydrocortisone in Oral Powder Form Compounded for Pediatric Patients in Japan

**DOI:** 10.3390/pharmaceutics13081267

**Published:** 2021-08-17

**Authors:** Jumpei Saito, Nozomi Yoshikawa, Takehisa Hanawa, Ayuna Ozawa, Takahiro Matsumoto, Tsutomu Harada, Kana Iwahashi, Hidefumi Nakamura, Akimasa Yamatani

**Affiliations:** 1 Department of Pharmacy, National Center for Child Health and Development, Setagaya-ku, Tokyo 157-0054, Japan; yoshikawa-no@ncchd.go.jp (N.Y.); iwahashi-k@ncchd.go.jp (K.I.); yamatani-a@ncchd.go.jp (A.Y.); 2 Division of Clinical Pharmacology and Oral Formulation Development, National Center for Child Health and Development, Tokyo 157-0054, Japan; 3 Faculty of Pharmaceutical Sciences, Tokyo University of Science, Chiba 278-8510, Japan; t-hanawa@rs.tus.ac.jp (T.H.); 3b18026@ed.tus.ac.jp (A.O.); 4 R&D Center, Ohara Pharmaceutical Co., Ltd., Koka-shi 520-3403, Japan; takahiro-matsumoto@ohara-ch.co.jp; 5 Division of Pharmaceutics, School of Pharmacy, Showa University, Tokyo 142-8555, Japan; tharada@pharm.showa-u.ac.jp; 6 Department of Research and Development Supervision, National Center for Child Health and Development, Tokyo 157-0054, Japan; nakamura-hd@ncchd.go.jp

**Keywords:** hydrocortisone, pediatric formulation, oral powder, stability, quality assurance

## Abstract

Hydrocortisone has been utilized in the management of adrenal insufficiency. For pediatric patients, the commercially available enteral form of hydrocortisone tablets (Cortoril^®^) is administered in powder form after being compounded by a pharmacist. However, the stability and quality of compounded hydrocortisone powder have not been verified. In this study, we formulated a 20 mg/g oral hydrocortisone powder by adding lactose monohydrate to crushed and filtered hydrocortisone tablets and assessed the stability and physical properties of this compounded product in polycarbonate amber bottles or coated paper packages laminated with cellophane and polyethylene. Stability was examined over 120 days in three storage conditions: closed bottle, in-use bottle, and laminated paper. Drug dissolution and powder X-ray diffraction analysis were conducted to assess its physicochemical stabilities. Validated liquid chromatography-diode array detection was used to detect and quantify hydrocortisone and its degradation products. Although impurity B (cortisone) and G (hydrocortisone-21-aldehyde) were found after 120 days of storage, no crystallographic and dissolution changes were noted. Hydrocortisone content was maintained between 90% and 110% of initial contents for 120 days at 25 ± 2 °C and 60 ± 5% relative humidity in all packaging conditions.

## 1. Introduction

Hydrocortisone is a corticosteroid indicated for replacement therapy in pediatric patients with adrenocortical insufficiency [1,2]. Guidance for this therapeutic strategy in this specific population is to administer the lowest possible dose and to use the native hormone, that is, hydrocortisone (cortisol), rather than synthetic steroids such as prednisolone and dexamethasone, which have a greater suppressive effect on growth [3]. The hydrocortisone dose range is 7.5–15 mg/m^2^/day, according to the primary diagnosis of adrenal insufficiency, given in three to four divided doses, with the first and slightly higher dose on waking in the morning and the last dose 4 to 6 h before bedtime.

In 2018, in the United States and European Union, the immediate-release granule formulation of hydrocortisone, Alkindi^®^ (Eton Pharmaceuticals, Deer Park, IL, USA), was approved for the treatment of pediatric adrenal insufficiency. This formulation contains 0.5, 1, 2, and 5 mg of hydrocortisone in each capsule [4]. Previously, in these countries, hydrocortisone was only licensed in 10 and 20 mg tablet doses; compounding by pharmacists or caregivers was required either by tablet crushing or using the hydrocortisone base to produce a powder or unique solutions [5,6,7]. It remains unknown whether a hydrocortisone formulation will be developed for pediatric patients and if compounding will continue to be necessary in the future. Currently, the practice of compounding hydrocortisone at a hospital is unlicensed and thus not subject to the degree of regulation and quality control mandated for a licensed drug manufacturer; therefore, it is not optimal for safe and accurate administration. Furthermore, inconsistent hydrocortisone concentration in compounded formulations can lead to severe clinical consequences with poor disease control due to either an inadequate dose [6] or hypercortisolism from overdose [8,9].

Based on survey results from 208 pediatric hospitals in Japan, about 20% of facilities used compounding to administer hydrocortisone tablets for pediatric patients [10]. Manufacturers do not provide stability data for compounded hydrocortisone tablets; therefore, the pharmacotherapeutic quality of compounded hydrocortisone products remains unguaranteed. According to the International Council for Harmonisation of Technical Requirements for Pharmaceuticals for Human Use (ICH) guidelines, hospitals should preferably implement physical property testing of active pharmaceutical ingredients, including a stability study and dissolution study [11]. Although an attempt was made to develop solid dosage forms (a mini-tablet form) of hydrocortisone for pediatric patients [12], to the authors’ best knowledge, the stability of hydrocortisone powder, prepared by crushing commercially available tablets, has not been assessed. Thus, this calls for an implementable standardized method to ensure the quality of compounded hydrocortisone at pediatric hospitals. Several studies suggested that storage under light, at enhanced temperature, and in the presence of moisture had pronounced degradative effects on the stability of solid steroid-related drug substances [13,14]. According to the product information [15], crushed Cortril^®^ powder form that stored at room temperature in the amber condition is stable at day 30 after tablet crushing, no stability study was conducted on the compounded product with lactose hydrate.

The objectives of this study were to formulate an oral powder form of hydrocortisone in extra-fine crystal lactose hydrate at a concentration of 20 mg/g and assess its stability and physical properties in three storage conditions: closed bottle, in-use bottle, and laminated paper.

## 2. Materials and Methods

### 2.1. Reagents and Test Solution Preparation

**Figure 1:**
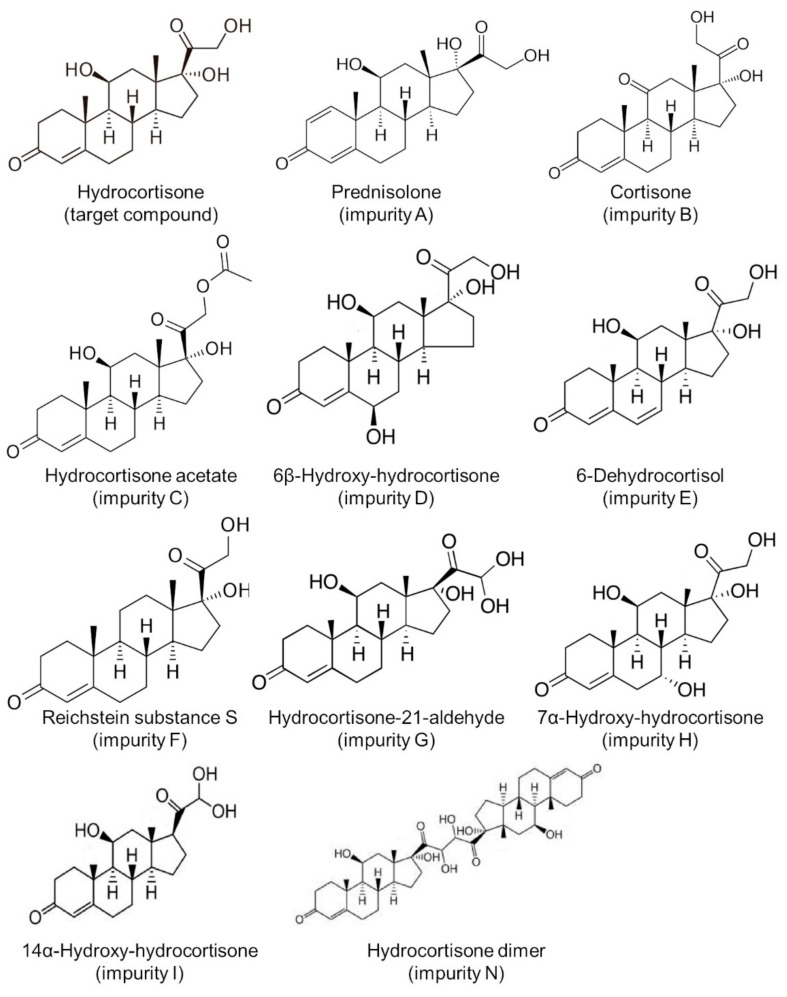
Structure of hydrocortisone and its impurities.

All chemicals and solvents were of analytical grade. Water for chromatography was obtained from a reverse osmosis system (Merck Millipore, Darmstadt, Germany). Hydrocortisone (target substance, purity > 99.0%) was purchased from Tokyo Chemical Industry (Tokyo, Japan). Impurities of hydrocortisone were procured from Sigma-Aldrich (Tokyo, Japan) as follows: prednisolone (impurity A), cortisone (impurity B), hydrocortisone acetate (impurity C), 6β-hydroxy-hydrocortisone (impurity D), 6-dehydrocortisol (impurity E), Reichstein substance S (impurity F), hydrocortisone-21-aldehyde (impurity G), 7α-hydroxy-hydrocortisone (impurity H), 14α-hydroxy-hydrocortisone (impurity I), and hydrocortisone dimer (impurity N) [16,17,18,19] (Figure 1). Lactose monohydrate (extra-fine crystal lactose hydrate “Hoei”, Pfizer, Tokyo, Japan) was used as a diluting agent. Standard solutions for hydrocortisone (100 μg/mL) and its impurities (100 μg/mL each) were prepared by dissolving 1 mg of the respective substances in 10 mL of 50% (*v*/*v*) methanol/water. Hydrocortisone test solutions were then prepared by dissolving 10 mg of stored compounded powders in 10 mL of 50% (*v*/*v*) methanol/water and then diluting with the solvent mixture to obtain final concentrations of 100 μg/mL. Test solutions were then prepared in triplicate.

### 2.2. Hydrocortisone Powder Compounding

Hydrocortisone in powder form was prepared in the Pharmaceutical Department of the National Center for Child Health and Development, according to the Regulations for Buildings and Facilities for Pharmacies. Crushing was performed for 500 × 10-mg Cortril^®^ tablets (Pfizer) (5000 mg of hydrocortisone) using an automatic pill crusher (KC-HUK2, Konishi Medical Instruments, Osaka, Japan) at 6000 rpm for 30 s. Cortril^®^ tablets contain potato starch, sucrose, precipitated calcium carbonate, hydroxypropyl cellulose, magnesium stearate, sodium lauryl sulfate, and carmellose sodium as inactive ingredients. Crushed tablets were then filtered using a Japanese Pharmacopoeia-certified No. 30 test sieve with 500 μm of nominal aperture (Tokyo Screen, Tokyo, Japan). Crushing and sieving were repeated until all of the samples passes through the sieve. Extra-fine crystal lactose hydrate was added to the sieved powder to make 20 mg/g hydrocortisone powder. An automatic mixer (YM-500, Yuyama, Tokyo, Japan) was used to mix the powder at 620 rpm for 60 s. Compounded hydrocortisone powders were placed in a stability chamber (SRH-32VEVJ2, Nagano Science Co., Ltd., Osaka, Japan) at 25 °C ± 2 °C/60% ± 5% relative humidity [11].

### 2.3. Stability Study

Hydrocortisone powder stability was assessed in samples drawn on days 0, 30, 60, 90, and 120, according to three schedules [20]: (1) the “bottle (closed)” condition, for which samples were drawn from distinct polycarbonate amber bottles (Yamayu, Osaka, Japan); (2) the “bottle (in-use)” condition, for which samples were drawn from one amber polycarbonate bottle, from which 0.1 g was removed daily in a clinical setting; and (3) the “laminated paper” condition, for which samples were drawn from a coated paper package laminated with cellophane and polyethylene (TK70W, Takazono, Tokyo, Japan). The change in drug content was calculated as (measured concentration/initial concentration) × 100 (%). Changes within 10% of the initial content were considered acceptable changes [21].

### 2.4. Dissolution Test

Dissolution tests were conducted according to the ICH Japanese Pharmacopoeia 17.6.10 (paddle method; NTR-6400AC; Toyama Sangyo, Tokyo, Japan) using 900 mL the dissolution medium maintained at 37 ± 0.5 °C and agitated at 50 rpm [22]. Distilled water was used as the dissolution medium, according to the test methods for the original tablet [15]. At each sampling time, 1.5 mL of the dissolution medium was withdrawn, filtered (0.22-μm syringe filter; Millipore, Darmstadt, Germany), and stored in test vials at −20 °C until analysis. The hydrocortisone powders obtained by crushing tablets were transferred into a dissolution vessel with wax paper. Six samples from each condition—closed bottle, in-use bottle, and laminated paper—collected after 0, 30, 60, 90, and 120 days of storage were analyzed using the liquid chromatography-diode array detection (LC-DAD, ThermoFisher Scientific K.K., Tokyo, Japan) method. The mean dissolution rate of each sample was compared with the sample compounded on day 0.

### 2.5. Powder X-ray Diffraction Analysis

Hydrocortisone powders stored in the closed bottle condition for 60, 90, and 120 days were subjected to powder X-ray diffraction (PXRD) analysis. The PXRD study was conducted using a RINT 2000 (Rigaku, Tokyo, Japan). The crystallinity of the obtained solid phase was measured at 40 kV voltage, 40 mA current, and a 4°/min scan rate with a nickel filter and a CuKα1 radiation source.

### 2.6. Assays of Hydrocortisone and Its Impurities

#### 2.6.1. Instrumentation and Chromatographic Conditions

A validated LC method was used to detect hydrocortisone and its impurities, as reported in a previous study [19]. An Ultimate 3000 HPLC system (Thermo Fisher Scientific, Tokyo, Japan), composed of an autosampler, column oven, and DAD was used. The autosampler was set at 10 °C, and the column oven was set at 40 °C. Chromatographic separation was performed on a C18 column (Imtakt US-C18 column; 150 × 3.0 mm, 5 μm; Imtakt, Kyoto, Japan). Eluent A was 10 mM ammonium formate in water, adjusted to pH 3.5 [23], and eluent B was acetonitrile. A gradient with two isocratic separation modes at a constant flow rate of 0.4 mL/min was then applied, from 0 to 20 min, and the composition of eluent B was maintained at 40%; then, a second isocratic step from 20 to 27 min was used with 60% of eluent B. From 27 to 32 min, the composition of eluent B was reduced to 40%. The eluents were then filtered through a 0.22-μm filter (Merck Millipore, Darmstadt, Germany). The injection volume was set at 5 μL. Detection was performed at 254 nm. Data acquisition, recording, and reprocessing were performed using Chromeleon software version 6.80 (Thermo Fisher Scientific).

#### 2.6.2. Linearity, Precision, and Accuracy

The response linearity was evaluated in triplicate at 6 concentrations ranging from 0.5 to 100 μg/mL for hydrocortisone (0.5, 1, 5, 10, 50, and 100 μg/mL) and 0.05 to 10 μg/mL for 10 hydrocortisone impurities (0.05, 0.1, 0.5, 1, 5, and 10 μg/mL). The calibration curves were validated to ensure that the sample concentrations were within the linear analyte response range. The standard calibration curve fitness was confirmed by back calculating the calibration standard concentrations. A weighted linear regression (weighting factor: 1/x^2^) method was used to obtain the standard calibration curve and the correlation coefficient. Calibration curve correlation coefficients greater than 0.99 are acceptable for determination using the LC-DAD system. Precision was then evaluated in terms of repeatability (intraday) and intermediate precision (inter-day). Results were expressed as the mean and relative standard deviation. Repeatability was then examined using six replicate injections of standard solutions, spiked at 100 μg/mL. The intermediate precision was evaluated by performing six replicate injections on three different days. The accuracy was calculated as (measured concentration/nominal concentration) × 100 (%). The limit of detection and the limit of quantification was calculated according to ICH guidelines [24].

### 2.7. Assay for Known and Unknown Hydrocortisone Impurities

Previously described hydrocortisone impurities (A, B, C, D, E, F, G, H, I, N) were identified with UV detection at 254 nm [17]. Their retention times were collected for potential identification and quantification during stability studies. An unknown hydrocortisone impurity was also examined. The contents of each impurity were evaluated by comparing the relative peak area of hydrocortisone.

## 3. Results

### 3.1. Liquid Chromatography Method and Validation

**Figure 2:**
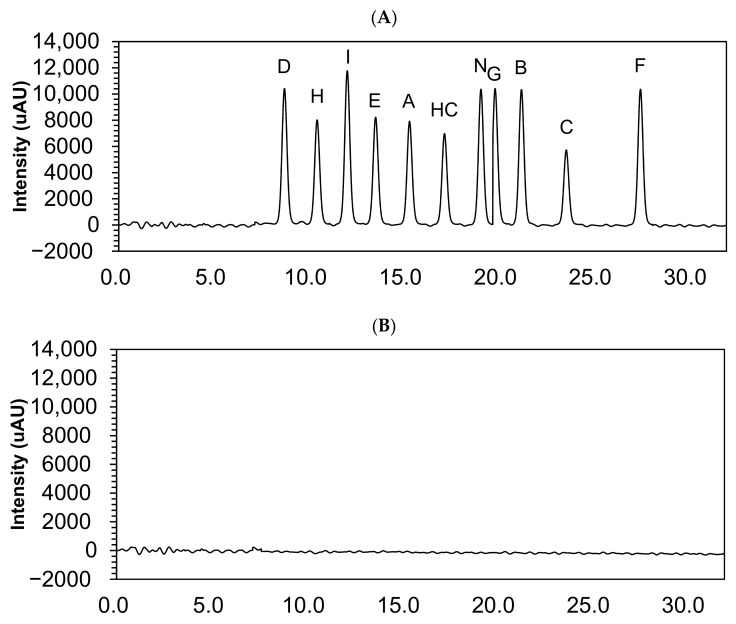
Chromatograms of 10 µg/mL hydrocortisone and its impurities in the solvent mixture (**A**) and solvent mixture (**B**). HC, hydrocortisone.

Hydrocortisone and its impurities retention times are shown in Table 1. The chromatograms showed no interfering peak eluting at the retention times (Figure 2).

Weighted linear regression analyses were also conducted, and linearity was observed over the examined concentration ranges. The regressions within this range had correlation coefficients greater than 0.99, indicating that the method provided a good linear response for hydrocortisone.

The regression line slopes and intercepts for hydrocortisone in the solvent mixture and the hydrocortisone dissolved solution did not significantly differ in the selected ranges (0.5–100 μg/mL for hydrocortisone and 0.05–10 μg/mL for 10 hydrocortisone impurities). The repeatability (intraday) expressed as % relative standard deviation was found to be less than 1.0% for hydrocortisone. The recovery was between 98.1% and 109.0%. The method was demonstrated to be sufficiently accurate considering the required specifications for hydrocortisone content (±10.0%).

### 3.2. Stability Study

**Figure 3:**
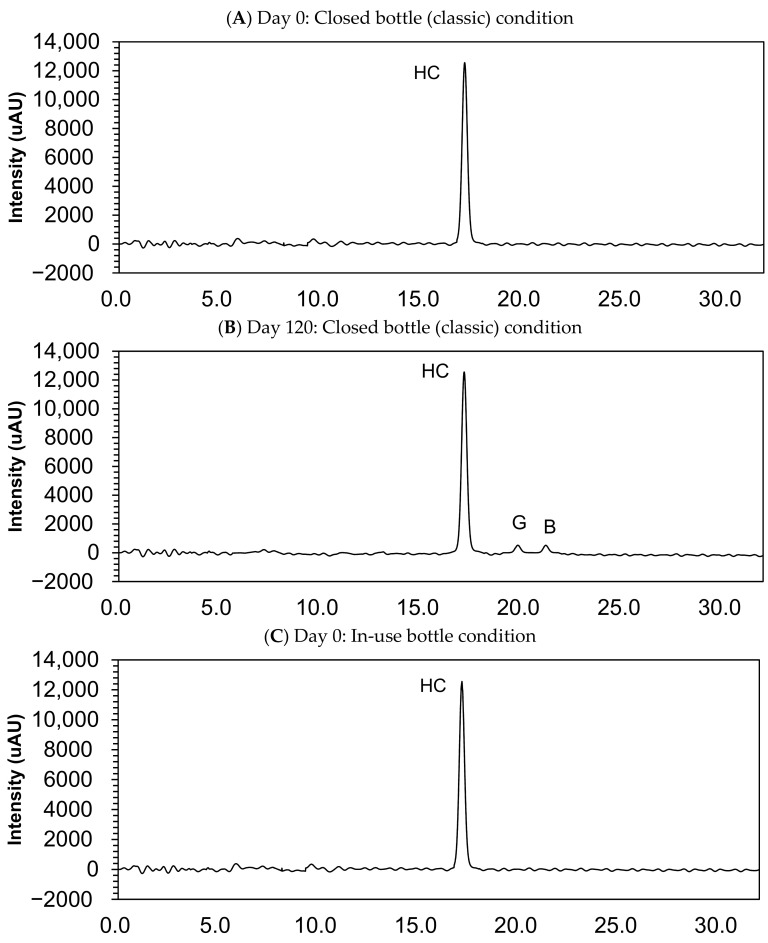

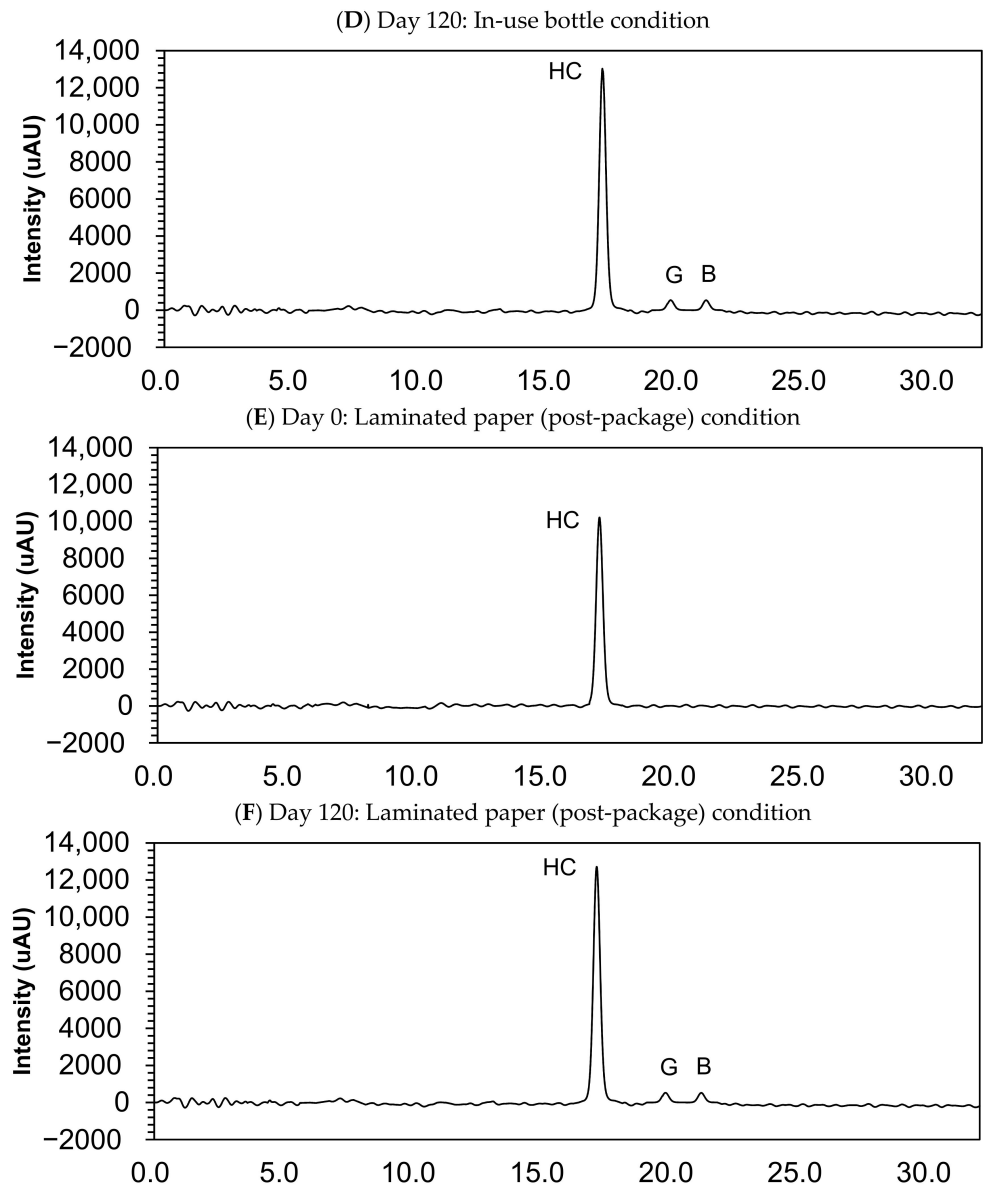
Chromatograms of compounded hydrocortisone in the closed bottle condition on day 0 (**A**) and day 120 (**B**), in the in-use bottle condition on day 0 (**C**) and day 120 (**D**), and in the laminated paper condition on day 0 (**E**) and day 120 (**F**). HC, hydrocortisone.

Table 2 and Figure 3 show the results of the stability study and the examples of chromatograms obtained for analysis of compounded hydrocortisone powders at day 0 and 120 at 25 °C ± 2 °C/60% ± 5% relative humidity in each three-sampling schedule. In the examined storage conditions, the hydrocortisone content remained within the specifications of 90.0–110.0% of the initial concentration during 120 days in each condition: closed bottle, in-use bottle, and laminated paper.

### 3.3. Dissolution Test

**Figure 4:**
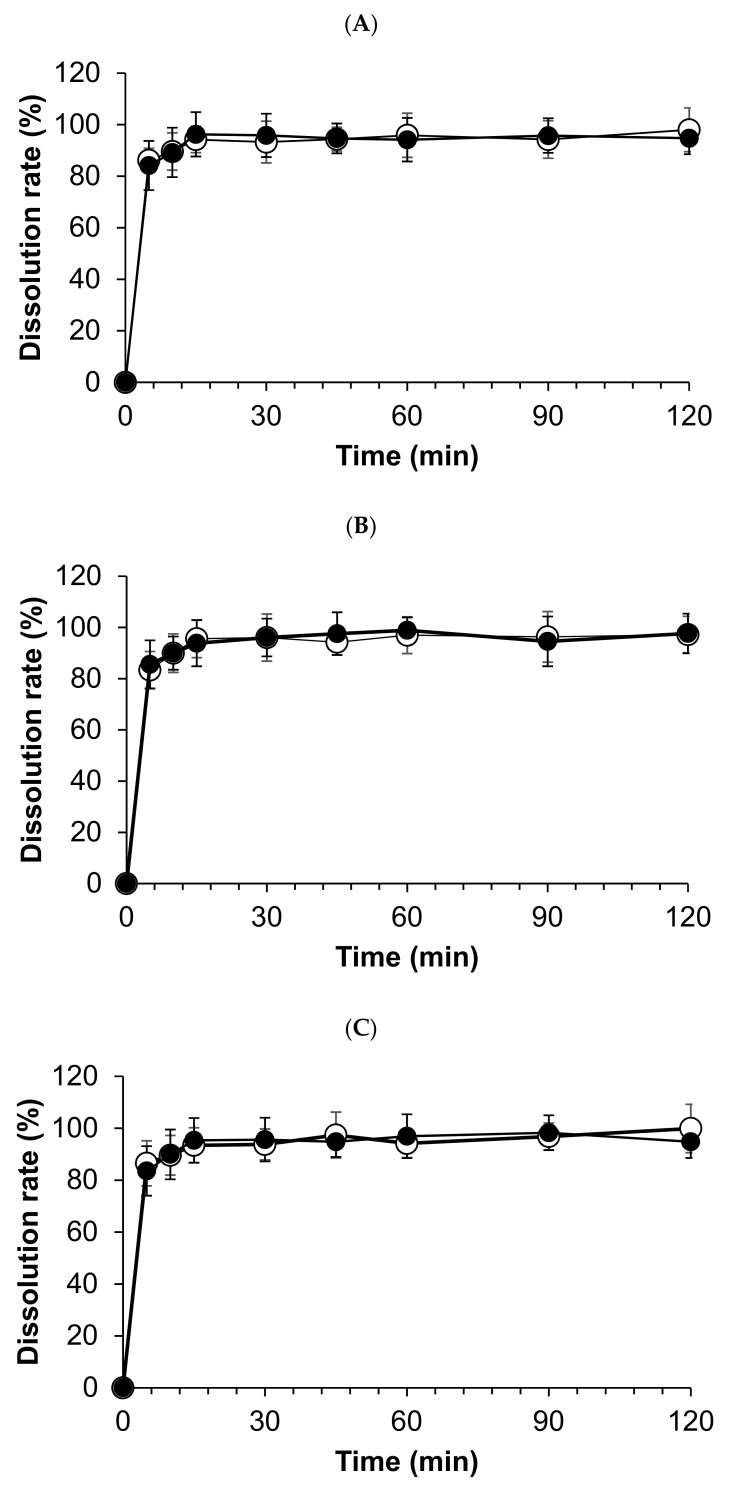
Dissolution profiles of compounded hydrocortisone formula in water stored in three conditions: closed bottle (**A**), in-use bottle (**B**), and laminated paper (**C**). Open circles indicate the dissolution rate on day 0; closed circles indicate the dissolution rate on day 120.

All compounded powders tested in distilled water have exhibited prompt and complete hydrocortisone dissolution, and the total amount of hydrocortisone dissolved in 15 min. However, dissolution profiles were not significantly observed among stored compounded forms and the crushed tablets at day 0 (Figure 4).

### 3.4. Impurity Study

Small quantities of impurity B (cortisone) and G (hydrocortisone-21-aldehyde) were found in the sample collected at day 120, but this quantity was deemed lower than 0.05%, and none of the other impurities were determined in the compounded powders (Figure 3).

### 3.5. PXRD Analysis

**Figure 5:**
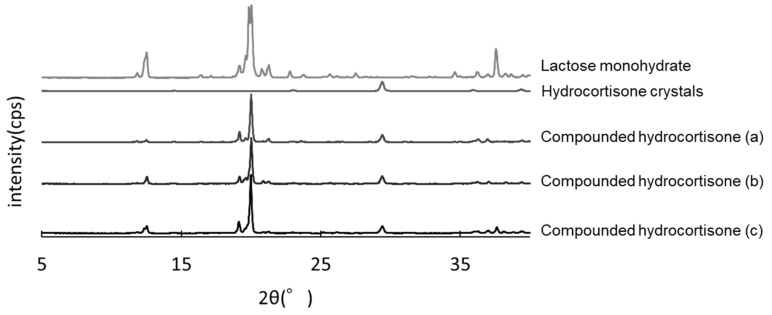
Powder X-ray diffractometry of hydrocortisone stored in the closed bottle condition for 60 days (a), 90 days (b), and 120 days (c).

The hydrocortisone crystals showed characteristic peaks at *2θ* = 29.5°. The same peaks were observed in compounded hydrocortisone powders stored in the closed bottle condition for 60, 90, and 120 days, showing no crystallographic changes over the storage period (Figure 5).

## 4. Discussion

Many commercially available enteral formulations are not designed for pediatric patients; therefore, adult formulations are often compounded by pharmacists in the hospital and community pharmacy setting or by caregivers at home [10], resulting in off-label and unlicensed administration of medicine for pediatric patients. Although several stakeholders have already expressed their support in developing pediatric formulations [25,26,27,28,29,30,31], an urgent need exists to improve the standardization in compounded enteral formulations to increase the safety and compliance of preparations made in Japan. To date, most tested hydrocortisone formulations for pediatric patients are liquids [17,32]; however, the stability and safety of solid oral compounded products have not been reported, except for hydrocortisone mini-tablet forms [12].

Regarding the stability of the hydrocortisone liquid formulation, Fawcett et al. demonstrated the 90-day stability of a 2-mg/mL hydrocortisone solution stored in the refrigerator [23]. This oral suspension was made with either tablets or powder and a vehicle containing sodium carboxymethylcellulose (1 g), syrup BP (10 mL), hydroxybenzoate 0.1% preservatives (0.1 g), polysorbate 80 (0.5 mL), citric acid (0.6 g), and water. Chong et al. also demonstrated 90-day stability (more than 90%) of hydrocortisone in tablet-dissolved solutions kept at room temperature [33]. This oral suspension was made from 10 mg tablets and a 1:1 mixture of Ora-Sweet (Medisca, Plattsburgh, NY, USA) and Ora-Plus (Perrigo, Perth, Australia). The stability over 90 days in 2 mg/mL of hydrocortisone at 25 °C created from hydrocortisone tablets and Oral Mix (Medisca) was also reported [34]. The 60-day stability of hydrocortisone 1 mg/mL oral suspension prepared from tablets and stored at room temperature in amber glass/plastic polyethylene terephthalate bottles was also reported in a monograph prepared for the Hospital for Sick Children in Canada [35].

Based on routine clinical practice in Japan, lactose hydrates or cornstarch are used as a typical diluting agent, although not used in other countries. In this context, information is needed on the stability of the compounded products diluted by these agents.

The impurity assay showed that impurity B (cortisone) and G (hydrocortisone-21-aldehyde) were found after 120 days of storage. This observation was similar to the findings reported by Wollmer et al. [17]. They have also found small concentrations of impurity B and G, all of which were lower than the USP and EP limits. Furthermore, none of the other impurities were determined after mixing the hydrocortisone granules with water.

To the best of our knowledge, this investigation is the first stability study of a 20 mg/g hydrocortisone oral powder that was prepared from commercially available hydrocortisone tablets and extra-fine crystal lactose hydrate. Based on the results from the stability test, the hydrocortisone content did not decrease, and hydrocortisone-related impurities were determined to be lower than 0.05% in the LC-DAD assay. These results also suggested that no adsorption of hydrocortisone occurred on the polycarbonate container or on the package. This study was conducted under the closely monitored storage conditions after hydrocortisone compounding. Real-world clinical settings have a risk of fluctuating storage conditions for compounded drugs, and further study on the stability tests under the loose condition that reflects the actual clinical settings will be required. A clinical pharmacist should be aware of this limitation, and compounded hydrocortisone should be stored under the appropriate condition. The study on the structural change by spectroscopic characterization after tablet compounding and explore which compounding methods are the most appropriate are also required. In addition, this study did not include treatment outcomes and adverse effects experienced by the patients, further evaluation of drug efficacy and safety is needed.

The authors of this study believe that this established compounding method guarantees the quality of pediatric hydrocortisone formula and may contribute to the standardization of hydrocortisone compounding for hospitals in Japan.

## 5. Conclusions

Hydrocortisone powder prepared from commercially available tablets remains stable for 120 days at 25 °C ± 2 °C and 60% ± 5% relative humidity in closed bottles, bottles opened daily, and packaged storage conditions. Since the results obtained in this study were investigated under certain controlled storage conditions, more attention should be paid to storage management in clinical practice.

## Figures and Tables

**Table 1:** Retention times for each compound.

	Compound Name	Retention Time (min)
Target compound	Hydrocortisone	17.1
Impurity A	Prednisolone	15.3
Impurity B	Cortisone	21.2
Impurity C	Hydrocortisone acetate	23.6
Impurity D	6β-Hydroxy-hydrocortisone	8.7
Impurity E	6-Dehydrocortisol	13.5
Impurity F	Reichstein substance S	27.5
Impurity G	Hydrocortisone-21-aldehyde	19.8
Impurity H	7α-Hydroxy-hydrocortisone	10.5
Impurity I	14α-Hydroxy-hydrocortisone	12.1
Impurity N	Hydrocortisone dimer	19.1

**Table 2:** Compounded hydrocortisone stability.

Study Methods	Storage Conditions	Storage Container	Test Periods (Days)				
			0	30	60	90	120
			Hydrocortisone Concentrations *				
Bottle (closed)	25 °C ± 2 °C/60% ± 5% relative humidity	Amber/PC bottle	100.0%	99.4 ± 2.2%	99.8 ± 4.1%	98.9 ± 4.1%	98.9 ± 3.6%
Bottle (in use)		Amber/PC bottle	100.0%	98.2 ± 1.3%	100.8 ± 1.7%	97.9 ± 3.8%	98.5 ± 2.2%
Laminated paper		Amber/CP laminated paper	100.0%	99.6 ± 1.2%	99.7 ± 3.4%	102.1 ± 2.4%	100.2 ± 3.1%

PC, polycarbonate; CP, cellophane and polyethylene. * Hydrocortisone concentrations are presented as percentages of the day 0 content (100%).

## Data Availability

Not applicable.
